# Integrated physiological and transcriptomic analyses elucidate the molecular mechanisms of exogenous melatonin-mediated salt tolerance in pomegranate (*Punica granatum* L.)

**DOI:** 10.3389/fpls.2025.1658091

**Published:** 2025-10-30

**Authors:** Yuanling Yang, Huiying Liu, Haosheng Gai, Yang Liu, Mingxuan Liu, Linnan Wu, Ming Diao

**Affiliations:** Key Laboratory of Special Fruits and Vegetables Cultivation Physiology and Germplasm Resources Utilization of Xinjiang Production and Construction Corps, Department of Agricultural College of Shihezi University, Shihezi, China

**Keywords:** melatonin, salt stress, pomegranate, transcriptomics, physiology, WGCNA analysis

## Abstract

Salt stress is a critical constraint affecting the cultivation of Tunisian soft-seeded pomegranate (*Punica granatum* L.). To elucidate the molecular mechanisms underlying exogenous melatonin (MT)-mediated enhancement of salt tolerance in pomegranate seedlings, this study integrated physiological phenotyping and transcriptome sequencing to systematically investigate MT’s regulatory effects on antioxidant systems, photosynthetic apparatus function, osmotic adjustment, and core metabolic pathways under salt stress. The results demonstrated that 200 mM NaCl treatment induced reactive oxygen species (ROS) overaccumulation, elevating malondialdehyde (MDA) content and relative electrical conductivity (REC) by 0.43 and 0.46 fold, respectively. Concurrently, salt stress severely impaired photosynthetic performance: PSII maximum photochemical efficiency (F_V_/F_M_) decreased by 44.5%, actual photochemical efficiency (Y_II_) and photochemical quenching (qP) were reduced, and non-photochemical quenching (NPQ) increased, indicating serious photoinhibition and energy wastage. In contrast, 400 μM MT treatment effectively mitigated oxidative damage by coordinated activation of superoxide dismutase (SOD,+14.3%), peroxidase (POD,+21.7%), and catalase (CAT,+11.7%) activities, thereby stabilizing membrane integrity. Furthermore, MT significantly alleviated photoinhibition: *F*_V_/*F*_M_ increased by 39%, Y_II_ and qP rose, and NPQ decreased compared to salt-stressed plants, reflecting enhanced protection of the PSII reaction center and optimized light energy allocation. Transcriptomic analysis reveals that MT treatment is associated with alterations in the expression of key sucrose metabolism genes, including the upregulation of *SUCROSE SYNTHASE* (*SUS*) and *UDP-GLUCOSE PYROPHOSPHORYLASE* (*UGP2*), as well as the recovery of *GLYCOGEN SYNTHASE* (*glgA*) expression following salt stress inhibition. These changes suggest a potential role for MT in modulating carbon metabolic homeostasis. Additionally, MT application is linked to expression changes in genes within the Mitogen-Activated Protein Kinase (MAPK) signaling pathway. Concurrently, broad expression variations are observed in genes associated with multiple phytohormone signaling pathways. Weighted gene co-expression network analysis (WGCNA) further identifies two core gene modules: the blue module is enriched with antioxidant-related genes (e.g., *LOC116212144*), while the yellow module is closely associated with genes implicated in membrane stability (e.g., *LOC116203737*). Integrated physiological and transcriptional evidence indicates that exogenous melatonin may enhance salt tolerance in pomegranate seedlings by activating the antioxidant system, protecting photosynthetic apparatus, regulating carbon metabolism, and influencing multiple signal transduction pathways. This study provides a theoretical foundation for further elucidating the mechanistic basis of MT-mediated salt adaptation in plants.

## 1 Introduction

Soil salinization has emerged as a critical environmental challenge threatening global agricultural productivity. Globally, over 900 million hectares of land are affected by excessive salt accumulation (Zhou et al., 2024), of which approximately one-third is irrigated cropland. In China, accelerated agricultural modernization and land exploitation have exacerbated soil salinization, with affected areas expanding annually, significantly constraining crop production (Zhuang et al., 2021). The issue is particularly severe in northwestern, northern, and northeastern China, where arid and semi-arid conditions exacerbate salt-induced degradation, compromising agricultural sustainability. Fruit tree cultivation is subject to disproportionate impacts, with salt stress triggering leaf chlorosis, wilting, yield reduction, and growth inhibition (Sinha et al., 2022). These physiological disruptions directly compromise farmers’ economic returns and market competitiveness of horticultural products.

The Tunisian soft-seed pomegranate (*Punica granatum* L.), which has recently been introduced into China as a high-quality economic fruit tree, has gained considerable consumer appreciation due to its soft seeds, excellent palatability, and rich nutritional profile (Liu et al., 2023). Nevertheless, its cultivation in saline-affected regions is accompanied by considerable challenges, resulting in severe restrictions on its widespread adoption in salt-degraded soils (Singh et al., 2023). The impact of salt stress on plant physiology is primarily characterized by two overarching mechanisms: ionic toxicity and osmotic stress. These mechanisms collectively result in the excessive accumulation of reactive oxygen species (ROS). This oxidative burst has been shown to cause significant damage to cellular structure and function, with membrane stability being particularly vulnerable (Liu et al., 2019; Nawaz et al., 2023). The overproduction of ROS initiates a cascade of oxidative injuries, including lipid peroxidation, protein denaturation, and DNA damage, which result in significant reductions in photosynthetic efficiency and metabolic activity. Consequently, the development of effective strategies to mitigate the impact of salt stress impacts on Tunisian pomegranate stands is a pressing scientific priority for sustainable horticulture in saline environments.

In recent years, melatonin (MT), a naturally occurring plant signaling molecule, has demonstrated significant potential in mitigating physiological damage induced by salt stress (Khan et al., 2022). Although initially believed to function exclusively in animals, MT has been established as essential in plant systems (Xie et al., 2022). MT influences plant growth, development, and responses to abiotic stresses through various pathways. It safeguards plant cells by enhancing the activities of antioxidant enzymes, such as superoxide dismutase (SOD), peroxidase (POD), and catalase (CAT), which are crucial for scavenging excess ROS (Zhang et al., 2015; Li et al., 2025). Furthermore, MT enhances osmotic regulation by promoting proline accumulation, thereby reducing membrane lipid peroxidation and maintaining cellular stability, as well as water and ion balance under salt stress conditions (Singh et al., 2023). These mechanisms underscore the significant potential of MT in mitigating salt stress and improving plant salt tolerance.

The application of exogenous MT has been demonstrated to significantly enhance salt tolerance across various crops (Hao et al., 2024; Himanshu et al., 2024; Khan et al., 2024). In rice, for instance, MT treatment has been shown to promote root growth, optimizes ion balance, and alleviate the detrimental effects of salt stress on photosynthesis (Yan et al., 2021). Similarly, in maize, MT has been shown to mitigate the adverse impact of salt stress on photosynthetic efficiency by activating secondary metabolic pathways, thereby augmenting plant biomass (Zhu et al., 2024a). Furthermore, MT has been shown to enhance stress resistance when combined with other plant hormones such as abscisic acid and gibberellins, thereby improving plant adaptability under stress conditions (Samanta and Roychoudhury, 2023; Cai et al., 2025). As demonstrated in our previous research, the application of 400 μM MT has been shown to enhance the salt tolerance of pomegranate by modulating the activities of SOD, POD, and CAT under 200 mM salt stress. However, research on the role of MT in pomegranate remains in its early stages, particularly concerning the molecular mechanisms underlying the response to salt stress, and comprehensive studies are still needed.

In this investigation, Tunisian soft-seeded pomegranate seedlings were employed as the primary research subjects. Through the integration of physiological phenotyping and transcriptome sequencing, we conducted a comprehensive examination of the molecular mechanisms by which exogenous MT mitigates salt stress. Our findings indicate that MT significantly diminishes lipid peroxidation and membrane permeability damage by activating antioxidant enzymes, including SOD, POD, and CAT, as well as by upregulating osmotic regulation genes such as *TREHALOSE-6-PHOSPHATE SYNTHASE* (*TPS*) and *SUCROSE SYNTHASE* (*SUS*). Utilizing weighted gene co-expression network analysis (WGCNA), we identified gene clusters associated with antioxidant capacity (blue module) and membrane stability (yellow module), along with key genes such as *LOC116212144* and *LOC116203737*. The findings of this study provide a theoretical foundation for enhancing salt tolerance in pomegranates and offer valuable insights for developing stress-resistance strategies in other horticultural crops.

## 2 Materials and methods

### 2.1 Experimental design

The experiment was conducted in the glass greenhouse of the North Garden New District at Shihezi University. The experimental material was 1-year-old pomegranate seedlings of the Tunisian soft-seeded variety (introduced from Henan Xingyang). The seedlings were planted in cultivation bags with a diameter of 14cm and a height of 16cm, using a substrate mixture of vermiculite: perlite: peat (1:1:1, V/V/V).

We randomly selected seedlings with uniform growth for different treatments. The experiment had three treatments: (1) CK: no salt stress (Hoagland nutrient solution + foliar spray with distilled water); (2) S: salt stress treatment (Hoagland nutrient solution + 200 mM NaCl); (3) MS: salt stress + foliar spray with 400 μM MT. The concentrations of MT and salt stress were selected from preliminary experiments. MT was applied by foliar spray at 8:00 pm on the day of treatment, until both sides of the leaves were completely wet but no liquid dripped. We sprayed every two days for a total of three times. For NaCl treatment, it was directly added to the nutrient solution, and a saucer was placed under each pot to prevent salt loss. Each treatment had three replicates. On the 15th day of salt stress treatment, mature functional leaves were selected and quickly brought to the laboratory for measurement.

### 2.2 Measurement of chlorophyll fluorescence parameters

We Chlorophyll fluorescence parameters were measured using a chlorophyll fluorescence imaging system (MAX-Imaging-PAM) on the 15th day after stress treatment between 10:00 and 12:00 a.m. Prior to measurement, pomegranate seedlings were dark-adapted for 30 minutes. The following parameters were determined: PSII maximum photochemical efficiency (F_V_/F_M_), photochemical quenching coefficient (qP), non-photochemical quenching coefficient (NPQ), and actual photochemical efficiency (Y_II_). Each treatment comprised three replicates, with three functional leaves selected from one seedling per replicate.

### 2.3 Physiological index determination

SOD activity was measured using the nitroblue tetrazolium (NBT) photochemical reduction method. POD activity was measured via the guaiacol colorimetric method; CAT activity was determined using the hydrogen peroxide oxidation-reduction method. The REC was measured using the conductivity method (Wang et al., 2003). MDA content was determined using the thiobarbituric acid method. Proline (Pro) content was measured using the acidic ninhydrin colorimetric method (Xiao, 2020). Each physiological experiment was independently repeated three times.

### 2.4 Determination of endogenous MT

We used high - performance liquid chromatography (HPLC) for determination. We weighed about 0.2g of sample, added 1 mL of 50% methanol, ground it, and extracted it overnight. After centrifugation, we collected the supernatant. HPLC conditions were as follows: L 3000 HPLC instrument (Rigol), C18 reverse - phase column (250 mm×4.6 mm, 5 μm). Mobile phase (A:B = 4:6, A: methanol, B: 0.1% formic acid). Injection volume: 10 μL. Flow rate: 0.8 mL/min. Column temperature: 30°C. Run time: 45min. Excitation wavelength: 280 nm. Emission wavelength: 348 nm.

### 2.5 Transcriptome sequencing

Total RNA was extracted using the Tiangen Plant RNA Kit. RNA purity and integrity were assessed via Nanodrop and RNA-specific agarose gel electrophoresis. Strand-specific libraries were constructed using the TruSeq Stranded mRNA LT Sample Prep Kit (Illumina, USA). mRNA was purified with the AMPure XP system. Library quality was evaluated using an Agilent Bioanalyzer 2100, total concentration by Pico green, and effective concentration by qPCR. Sequencing was performed on the Illumina HiSeq 2500 platform by Shanghai Personal Biotechnology Co., Ltd.

### 2.6 Screening of differentially expressed genes

Low-quality data were filtered using FASTP to obtain clean reads. The reference genome index was built using HISAT2, and all paired-end clean reads were aligned to the reference genome using HISAT2. The reference genome used in this study was the *Punica granatum* L. assembly version ASM765513v2 (GCF_007655135.1) from the NCBI database, with the link provided as: https://ftp.ncbi.nlm.nih.gov/genomes/all/GCF/007/655/135/GCF_007655135.1_ASM765513v2. HTSeq was used to count the reads for each gene as the raw expression level, which correlates with the gene’s true expression level, length, and sequencing depth. Gene expression differences among samples were calculated using the FPKM (fragments per kilobase of transcript per million mapped reads) standard (**Supplementary Table S1**). DESeq was used for differential expression analysis between treatments. Differentially expressed genes (DEGs) were selected with |log_2_FoldChange| > 1 and *P*<0.05. Functional enrichment analysis of DEGs was performed using GO and KEGG databases.

### 2.7 Weighted gene co-expression network analysis

We constructed a co-expression network using WGCNA to link physiological traits and transcriptomic data, identifying modules of closely related genes. This connects gene modules to physiological information, aiding in exploring the relationship between gene networks and physiological traits.

### 2.8 Quantitative real-time PCR analysis

Eight genes were randomly selected for qRT-PCR analysis. The primer sequences used in this study are listed in **Supplementary Table S2**. Total RNA was isolated using an RNA extraction kit (Tiangen Biotech, Beijing, China). The FQ-RT Primer Mix (Tiangen Biotech, Beijing, China) was used to reverse-transcribe sample RNA into first-strand cDNA. RT-PCR was performed using Power qPCR PreMix (Jierui, Shanghai, China) under the following conditions: 10min at 95 °C, followed by 40 cycles of 10 s at 95 °C and 34 s at 60 °C. The 2^−ΔΔCT^ method was used to measure the relative expression levels of the detected genes, with three biological replicates for each sample (Livak and Schmittgen, 2001).

### 2.9 Data analysis

One-way ANOVA and Duncan’s test were performed using Microsoft Excel 2021 (Microsoft Corporation, Redmond, USA) and SPSS 26.0 (SPSS Inc., Chicago, IL, USA). Effects were considered significant at *p*<0.05. Origin 2024 (OriginLab, Northampton, USA) was used for graphing.

## 3 Results

### 3.1 Effects of exogenous MT on chlorophyll fluorescence parameters in pomegranate seedlings under salt stress

**Figure 1:**
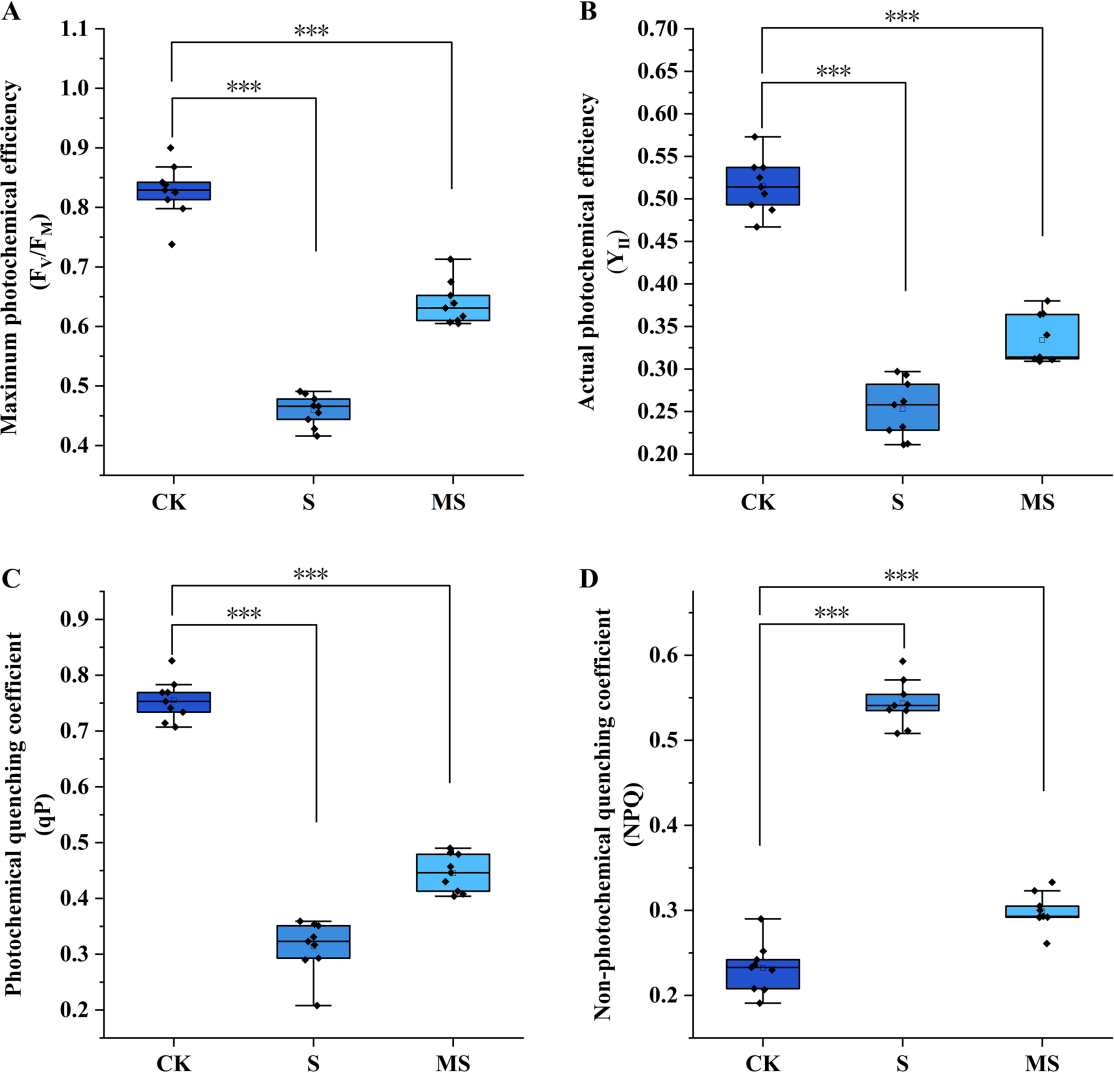
Effects of exogenous melatonin on chlorophyll fluorescence parameters in pomegranate seedlings under salt stress. **(A)** Maximum photochemical efficiency (F_V_/F_M_); **(B)** Actual photochemical efficiency (Y_II_); **(C)** Photochemical quenching coefficient (qP); **(D)** Non-photochemical quenching coefficient (NPQ). Asterisks denote significant differences compared to the control: *P<0.05, **P<0.01, ***P<0.001. Each experiment was repeated 3 times, and each sample in the experiments contained 3 biological replicates. The error bars in the graph represent standard deviation.

Chlorophyll fluorescence parameters are considered to be fundamental indicators of the functional state of the photosynthetic apparatus. In this study, the regulatory effects of salt stress and exogenous melatonin on the function of photosystem II (PSII) reaction centers in pomegranate seedlings were systematically analyzed by determining the maximum photochemical efficiency (F_V_/F_M_), the actual photochemical efficiency (Y_II_), the photochemical quenching coefficient (qP), and the non-photochemical quenching coefficient (NPQ) (**Figure 1**).

Under control (CK) conditions, the F_V_/F_M_ of pomegranate seedlings was maintained at 0.83 ± 0.04, within the normal range, indicating an intact structure and normal function of the PSII reaction centers. The Y_II_ reached 0.52 ± 0.03, reflecting a high level of actual photochemical efficiency where light energy was effectively utilized for CO_2_ fixation. A qP of 0.76 ± 0.04 suggested strong photochemical quenching, with the majority of absorbed light energy allocated to photochemical reactions. The NPQ was only 0.23 ± 0.03, indicating a moderate level of non-photochemical quenching where excess energy was dissipated via mechanisms such as thermal dissipation without damaging the photosynthetic apparatus. However, salt stress (S) treatment severely disrupted this balance. Compared to CK, the S treatment significantly reduced F_V_/F_M_ by 44.6% to 0.46 ± 0.02 (*P*<0.001), directly demonstrating severe damage (photoinhibition) to the PSII reaction centers. Y_II_ was also significantly reduced to 0.25 ± 0.03 (*P*<0.001), indicating a substantial decrease in actual photochemical efficiency and severe impairment of photosynthetic carbon assimilation capacity. Similarly, qP was significantly decreased to 0.31 ± 0.05 (*P*<0.001), reflecting weakened photochemical quenching, reduced openness of PSII reaction centers, and a marked decrease in the proportion of energy allocated to photochemical reactions. Concurrently, NPQ significantly increased to 0.54 ± 0.02 (*P*<0.001), indicating excessive enhancement of non-photochemical quenching. While this temporarily dissipated excess light energy to prevent further damage, the pronounced thermal dissipation also resulted in significant energy wastage, exacerbating the decline in photosynthetic efficiency.

Foliar application of exogenous melatonin (MS) significantly alleviated the salt stress-induced damage to the photosynthetic apparatus. Compared to the S treatment, the MS treatment significantly increased F_V_/F_M_ to 0.64 ± 0.03 (*P*<0.001). Although not fully restored to CK levels, this indicates a marked recovery in the structural integrity of the PSII reaction centers. Y_II_ was also significantly increased to 0.33 ± 0.03 (*P*<0.001), demonstrating improved actual photochemical efficiency and light energy utilization efficiency. qP increased dramatically to 0.45 ± 0.03 (*P*<0.001), indicating enhanced photochemical quenching, greater openness of PSII reaction centers, and increased allocation of light energy to photochemical reactions. Conversely, NPQ was significantly reduced to 0.30 ± 0.02 (*P*<0.001), returning to a level comparable to that of CK, suggesting that melatonin reduced excessive thermal dissipation and promoted more efficient energy utilization for photosynthesis.

In summary, salt stress severely inhibits photosynthetic function in pomegranate seedlings by disrupting the integrity of the PSII reaction center integrity, reducing photochemical efficiency, and inducing excessive energy dissipation. Conversely, exogenous melatonin significantly mitigates salt stress damage to the photosynthetic apparatus by protecting PSII reaction center integrity, enhancing photochemical efficiency, and optimizing energy allocation (increasing photochemical quenching while reducing non-photochemical quenching), thereby maintaining relatively stable photosynthetic function. These findings elucidate the key physiological pathway by which melatonin regulates salt tolerance in pomegranate seedlings and provide crucial support for subsequent molecular mechanism studies integrating transcriptomics.

### 3.2 Effects of MT on physiological indices of pomegranate seedling leaves under salt stress

**Figure 2:**
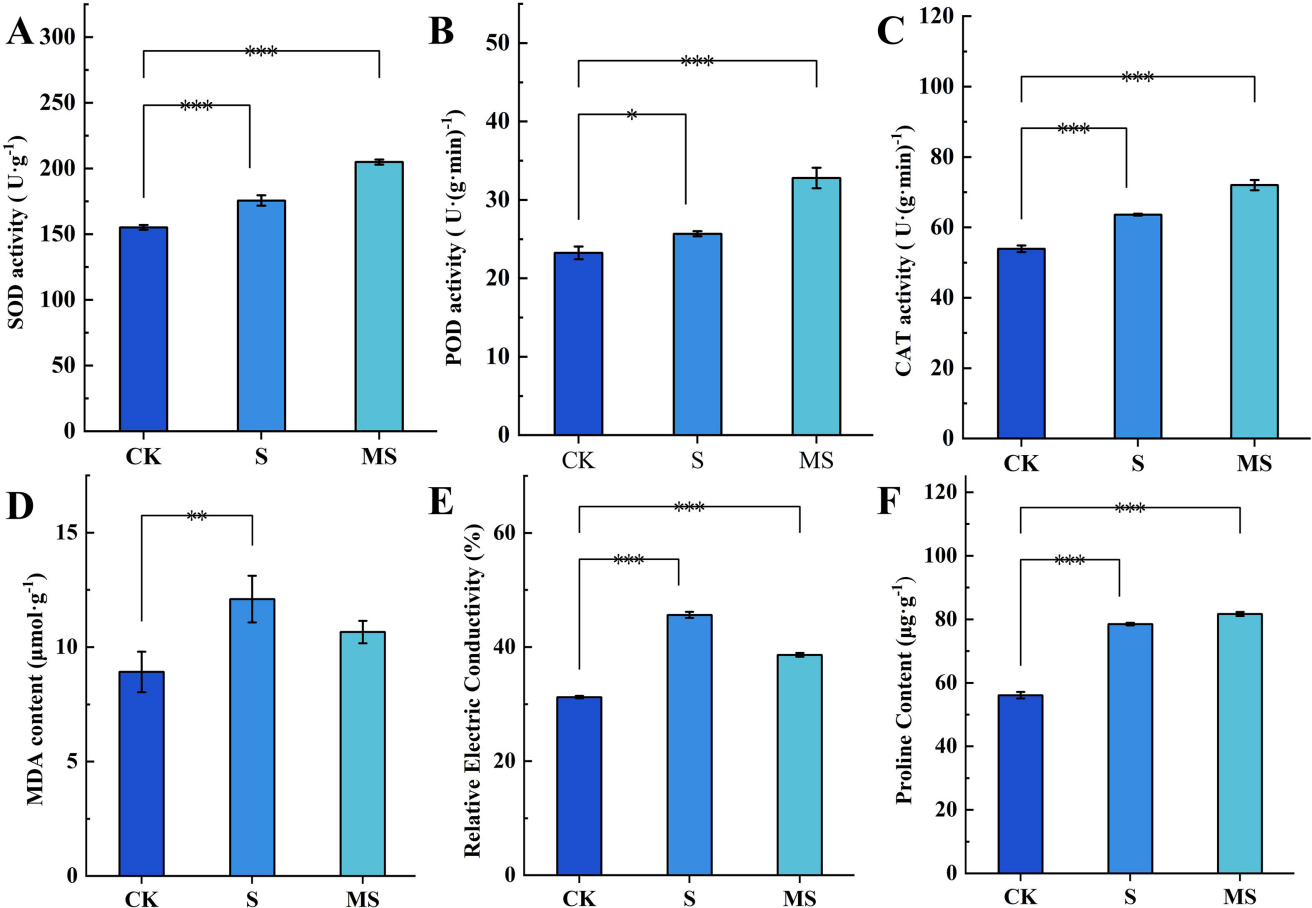
Effects of exogenous MT on physiological indexes of pomegranate seedling leaves under salt stress **(A)** SOD activity; **(B)** POD activity; **(C)** CAT activity; **(D)** MDA content; **(E)** REC values; **(F)** Pro content. Asterisks denote significant differences compared to the control: *P<0.05, **P<0.01, ***P<0.001. Each experiment was repeated 3 times, and each sample in the experiments contained 3 biological replicates. The error bars in the graph represent standard deviation.

Salt stress (S) significantly activated the antioxidant enzyme system in pomegranate seedlings (**Figures 2A–C**). Compared with the control group (CK), SOD (*P*<0.001) and POD (*P*<0.05) activities in the S group both increased by 0.16-fold, while CAT activity showed a significant 0.19-fold increase (*P*<0.001). These results indicate that plants counteract salt-induced oxidative stress by synergistically enhancing the activity of key antioxidant enzymes, thereby improving reactive oxygen species (ROS) scavenging capacity. Exogenous melatonin treatment (MS group) further enhanced this response: SOD, POD, and CAT activities increased by 14.3%, 21.7%, and 11.7% respectively, compared to the S group, demonstrating that melatonin significantly boosts ROS clearance efficiency by co-activating the antioxidant enzyme network.

Salt stress caused significant damage to cell membrane integrity (**Figures 2D, E**). In the S group, MDA content increased by 43% (*P < 0.01*), and REC increased by 46% (*P < 0.001*) compared to the CK group. This indicates that lipid peroxidation and membrane permeability imbalance are primary manifestations of salt damage. Melatonin treatment (MS) effectively reversed this trend: MDA content and REC were reduced by 16.42% and 18.1% respectively compared to the S group (*P*<0.001), suggesting that melatonin mitigates salt stress damage by inhibiting lipid peroxidation and stabilizing membrane structure.

Compared to the control group (CK), the proline content in the salt-stressed group (S) increased significantly by 40% (*P*<0.001)(**Figure 2F**). This result indicates that salt stress strongly activates proline synthesis in pomegranate seedlings. As a key adaptive response to osmotic stress, proline enhances cellular osmotic potential and mitigates water loss caused by ion toxicity. Notably, the proline content in the melatonin-treated salt-stressed group (MS) did not significantly differ from that in the S group, suggesting that melatonin did not suppress salt stress-induced proline accumulation but maintained its elevated expression.

### 3.3 Effect of MT on endogenous MT levels in pomegranate seedling leaves under salt stress

**Figure 3:**
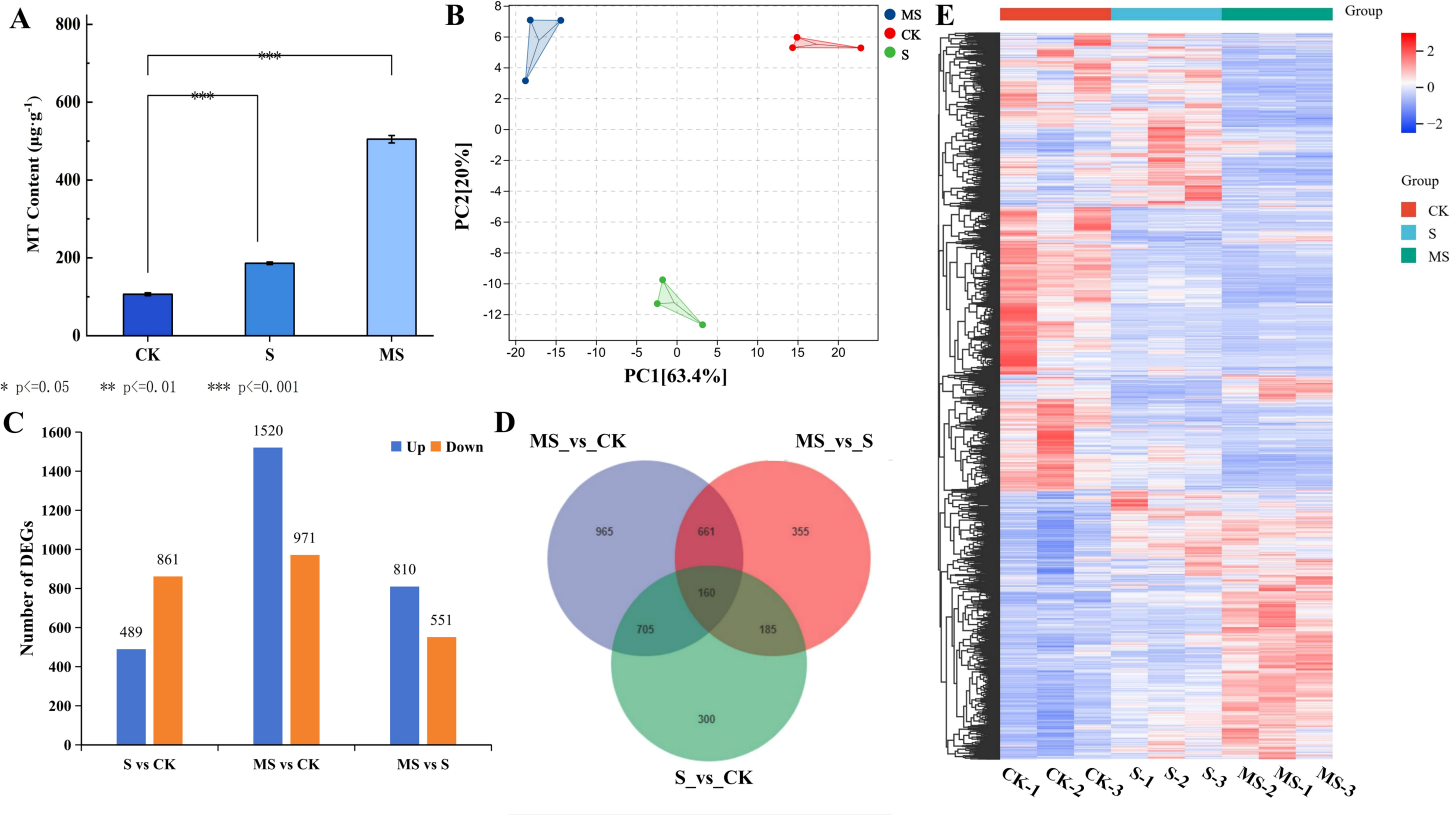
Changes in endogenous MT content and transcriptomics of pomegranate under different treatments **(A)** Changes in endogenous MT content under different treatments. Each experiment was repeated 3 times, and each sample in the experiments contained 3 biological replicates. The error bars in the graph represent standard deviation; **(B)** PCA correlation analysis; **(C)** Number of differentially expressed genes (DEGs) up- and down-regulated. **(D)** Venn diagram of shared DEGs. **(E)** Visualized heatmap of DEGs under different treatments.

In this experiment, endogenous MT was quantified in the leaves of pomegranate seedlings from different treatments using high-performance liquid chromatography (HPLC) (**Figure 3A**). Salt stress (S) significantly induced the accumulation of endogenous MT in pomegranate seedlings, and its content increased by 42.9% compared with the control group (CK) (S: 186.3 μg·g^-1^, CK: 106.4 μg·g^-1^, *P*<0.001). The significant increase in MT content under salt stress may be related to the endogenous stress response of plants. Exogenous melatonin treatment (MS) further dramatically increased the MT level to 505.0 μg·g^-1^, which was 78.9% and 63.1% higher than that of the CK and S groups, respectively, and the differences between the groups all reached highly significant levels (*P*<0.001). Exogenous MT may activate the endogenous synthesis pathway through positive feedback regulation, thus promoting endogenous MT synthesis.

### 3.4 Transcriptome analysis and identification of DEGs

In order to reveal the molecular mechanism of melatonin’s effect on pomegranate seedlings under salt stress, nine cDNA libraries were constructed by transcriptome assay analysis of pomegranate leaves from three different treatments. There were three biological replicates for each sample, and 68.22 Gb of clean data were obtained. Detailed information is shown in **Supplementary Table S3**. All samples had a percentage of Q20 bases > 97% and a percentage of Q30 bases > 93%. The filtered values of each sample were compared with the reference genome of pomegranate, and the comparison efficiency was > 99%, indicating that the quality of the obtained transcriptomic data was good. Principal component analysis (PCA) analysis revealed significant similarity between replicate samples in different treatments, indicating that the transcriptome data were reliable and reproducible (**Figure 3B**). The sequencing results were consistent with further analysis of the pomegranate transcriptome level.

A large number of differentially expressed genes were obtained based on comparative analysis of the three treatments, CK, S, and MS (**Supplementary Table S4**). heat maps of DEGs showed the different expression profiles of each treatment (**Figure 3E**). As can be seen in **Figure 3C**, a total of 1350 DEGs (489 genes up-regulated and 861 genes down-regulated) were screened in salt stress (S) compared with CK. In the MS vs CK comparison, the highest number of DEGs was screened with a total of 2491 DEGs (up-regulated 1520 genes and down-regulated 971 genes). In the MS vs S comparison, a total of 1361 DEGs were screened (810 genes up-regulated and 551 genes down-regulated). In the Wayne diagram (**Figure 3D**), the numbers of MS vs CK, MS vs S, and S vs CK-specific expressed genes were 965, 355, and 300, respectively (**Figure 2B**). This shows that more complex changes in genes within pomegranate leaves in response to salt stress occurred after exogenous spraying of MT.

### 3.5 GO enrichment and KEGG pathway analysis of differentially expressed genes

**Figure 4:**
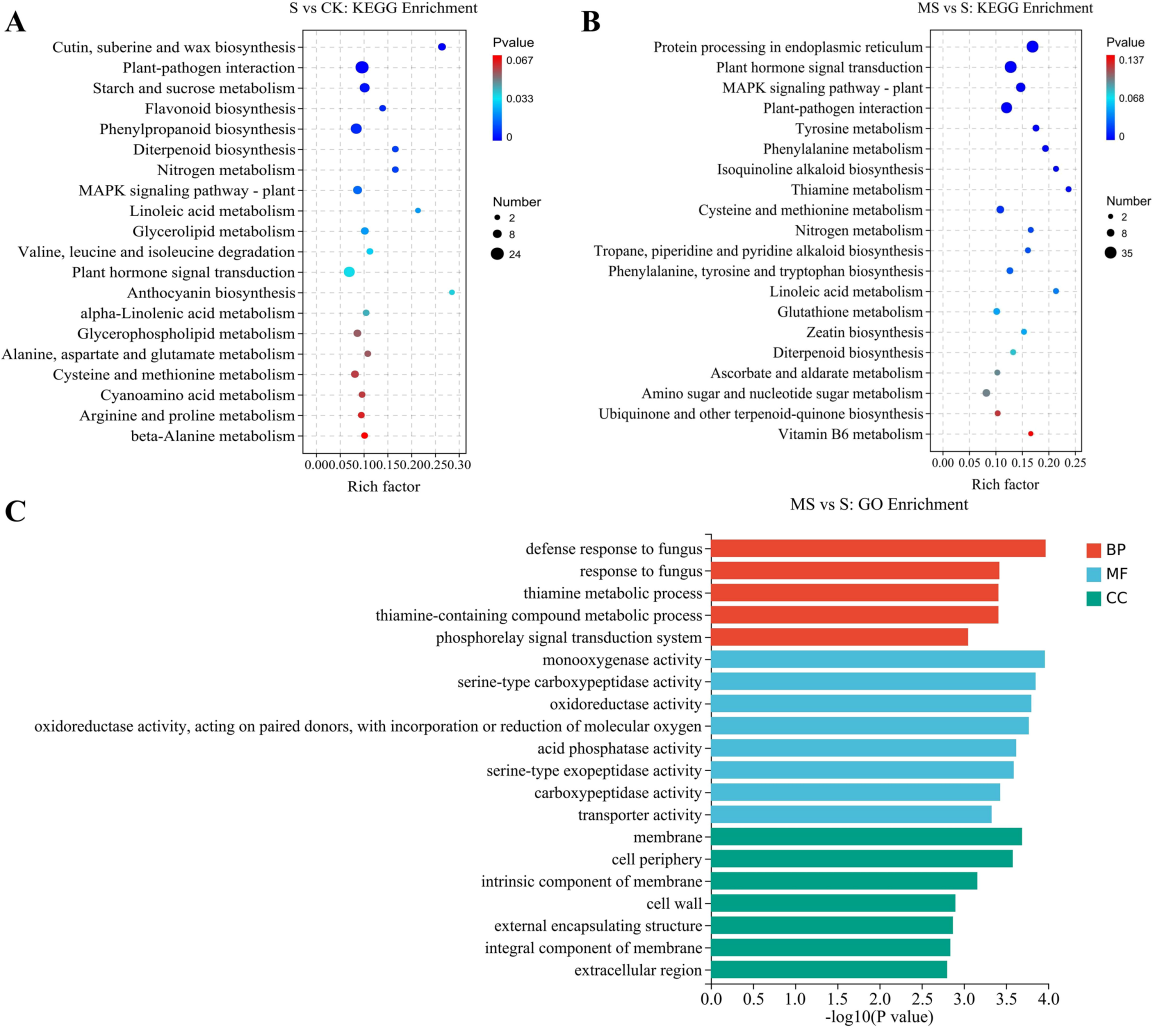
GO enrichment and KEGG pathway analysis of differentially expressed genes **(A)** GO enrichment of MS vs S comparison group.; **(B)** KEGG pathway analysis of S vs CK comparison group; **(C)** KEGG pathway analysis of MS vs S comparison group.

In order to explore more deeply the biological functions of DEGs in MT-treated pomegranate seedlings under salt stress, this study was conducted to functionally annotate the differentially expressed genes by GO enrichment analysis. This analysis was divided into 3 main aspects, describing the biological process (BP), molecular function (MF), and cellular component (CC) of the genes, respectively. In this study, to investigate the molecular regulatory mechanisms of MT on pomegranate seedlings under salt stress, we focused on the differentially expressed genes between MS vs S comparisons. The results showed that 1361 DEGs were enriched in the MS vs S comparison (**Figure 4A**), and the top 5, 8, and 7 GO terms of BP, MF, and CC were selected for display and analysis based on the number of DEGs. The GO enrichment in the BP category was mainly involved in the defense response, thiamine, and amine-containing compound metabolism. In MF, the categories with the highest GO enrichment involved oxidoreductase activity, acid phosphatase activity, serine exopeptidase activity, carboxypeptidase activity, and transporter activity. In the CC category, membranes, cell periphery, intrinsic components of membranes, cell wall, outer envelope parts, components of membranes, and extracellular regions were the major GO-enriched parts.

Genes often interact and will function together in certain metabolic pathways. In order to gain a comprehensive understanding of the biological pathways active in DEGs between treatments and to identify the major MT-related pathways. In this study, KEGG pathway enrichment analysis of differentially expressed genes was performed, focusing on up-regulated DEGs-enriched pathways in S vs CK and MS vs S comparisons. Important metabolic pathways were identified based on scatter plots combined with the number of DEGs (**Figures 4B, C**). There were 91 and 86 KEGG pathways significantly enriched in the S vs CK and MS vs S comparisons, respectively. As shown in **Figure 4B**, cutin, suberin and wax biosynthesis, plant-pathogen interactions, starch and sucrose metabolism, flavonoid biosynthesis, phenylketone biosynthesis, diterpene biosynthesis, nitrogen metabolism, the MAPK signaling pathway, linoleic acid metabolism, glycerol ester metabolism, valine, leucine, and isoleucine degradation, and phytohormone signaling were the metabolic pathways that were significantly enriched in the S vs CK comparison. Metabolic pathways such as protein processing, tyrosine metabolism, phenylalanine metabolism, isoquinoline alkaloid biosynthesis, thiamine metabolism, propane, piperidine, and pyridine alkaloid biosynthesis, and biosynthesis of phenylalanine, tyrosine, and tryptophan in the endoplasmic reticulum were unique and significantly enriched in the MS vs S comparison compared with S vs CK (**Figure 4C**). In contrast, pathways such as plant hormone signal transduction, MAPK signaling pathway, plant-pathogen interaction, cysteine and methionine metabolism, nitrogen metabolism, linoleic acid metabolism, and diterpenoid biosynthesis were co-significantly enriched in both the S vs CK and MS vs S comparisons (**Figures 4B, C**). These results suggest that the abovementioned highly conserved core pathways are likely key metabolic targets for MT-mediated regulation of salt tolerance in pomegranate seedlings.

### 3.6 DEGs involved in the plant hormone signal transduction

**Figure 5:**
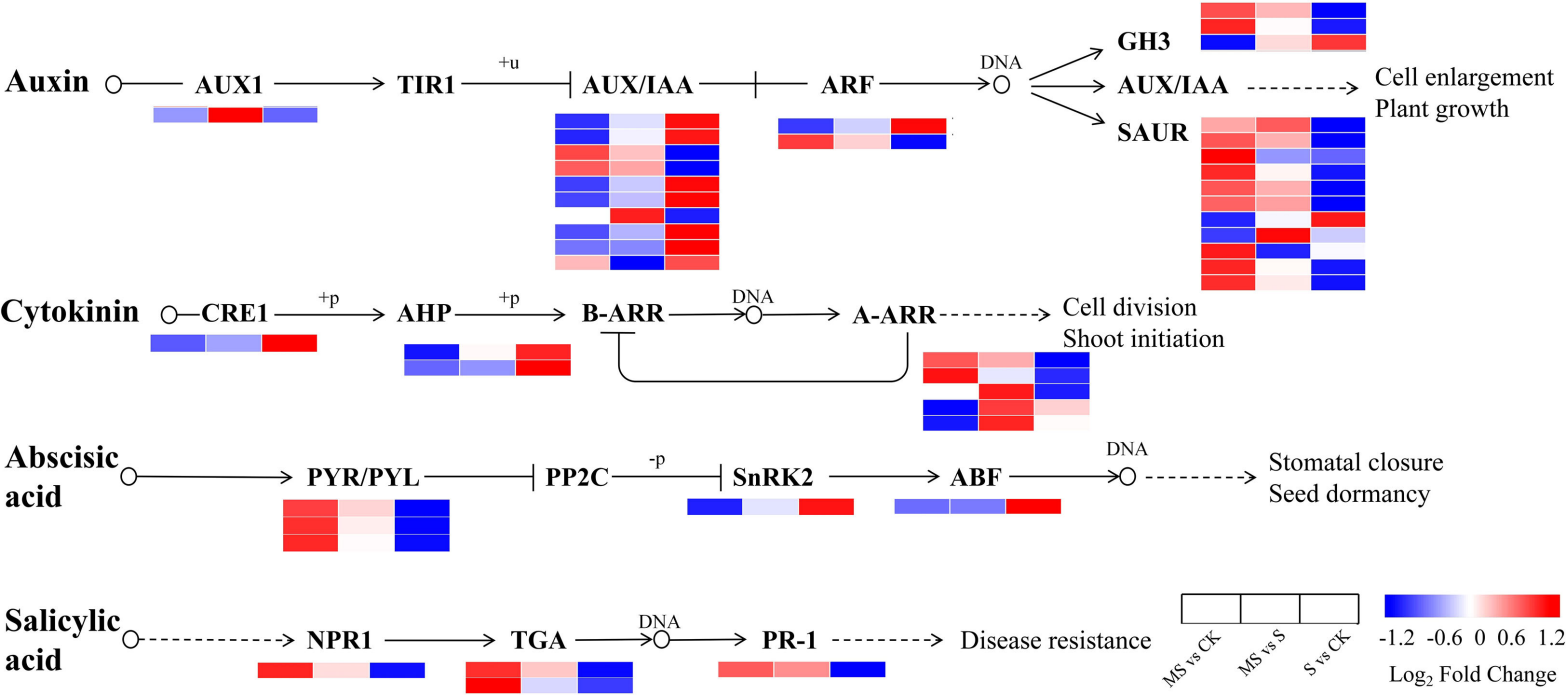
Schematic representation of DEGs involved in phytohormone metabolic pathways. Heatmaps were generated by log_2_FC. Changes in expression levels are indicated by color changes. Blue color indicates lower expression level while red color indicates higher expression level.

The plant hormone signal transduction pathway was found to produce 44 DEGs, including genes related to *AUXIN* (*IAA*), *CYTOKININ* (*CTK*), ABSCISIC ACID (*ABA*), and *SALICYLIC ACID* (*SA*) (**Figure 5**, **Supplementary Table S5**). In the growth hormone signaling pathway, the expression patterns of genes *AUXIN RESISTANT 1* (*AUX1)*, *SMALL AUXIN-UP RNA* (*SAUR)*, *GRETCHEN HAGEN 3* (*GH3)*, *AUXIN/INDOLE-3-ACETIC ACID* (*AUX/IAA)*, and *AUXIN RESPONSE FACTOR* (*ARF*) showed significant differences under different treatment conditions. In the S vs CK comparison group, the expression of one *AUX1*, nine *SAUR*, and two *GH3* genes was significantly down-regulated, suggesting that auxin signaling is likely suppressed by salt stress. In the comparison group of MS vs CK, the expression of three *AUX/IAA*, one *ARF*, nine *SAUR*, and two *GH3* genes was up-regulated, indicating an association between melatonin treatment and the activation of the auxin signaling pathway. Regarding the cytokinin signaling pathway, *CYTOKININ RESPONSE 1* (*CRE1)* and *ARABIDOPSIS HISTIDINE PHOSPHOTRANSFER* (*AHP)* protein genes were significantly up-regulated in the S vs CK comparison group, yet significantly down-regulated in the MS vs CK comparison group. Genes encoding *A-TYPE ARABIDOPSIS RESPONSE REGULATOR* (*A-ARR*) were upregulated by two and four in the MS vs CK and MS vs S groups, respectively, and three were downregulated in the S vs CK group. Collectively, these complex expression changes indicate that melatonin profoundly influences cytokinin signal transduction. In abscisic acid signaling, in the MS vs CK group, *PYRABACTIN RESISTANCE/PYRABACTIN RESISTANCE-LIKE* (*PYR/PYL)* showed up-regulation, and *SUCROSE NON-FERMENTING 1-RELATED PROTEIN KINASE 2* (*SnRK2)* and *ABA-RESPONSIVE ELEMENT BINDING FACTOR* (*ABF)* showed down-regulation. Conversely, *PYR/PYL* expression is reduced in S vs CK. These results suggest that melatonin participates in the stress response by modulating the expression of key components in the ABA signaling pathway. In the salicylic acid signaling pathway, *NONEXPRESSER OF PR GENES 1* (*NPR1)*, *TGACG MOTIF-BINDING TRANSCRIPTION FACTOR* (*TGA*), and *PATHOGENESIS-RELATED PROTEIN 1* (*PR-1)* showed up-regulation in the MS vs CK group but down-regulation in the S vs CK group. This expression pattern implies that melatonin treatment activates the SA-mediated defense response. By comparing the expression patterns of DEGs in MS, S, and CK groups, it was evident that exogenous melatonin significantly affected multiple phytohormone signaling pathways under salt stress conditions. These findings indicate that melatonin likely plays an important role in enhancing plant salt tolerance through the coordinated regulation of IAA, CTK, ABA, and SA signal transduction.

### 3.7 Key DEGs involved in starch and sucrose metabolic pathways and MAPK signaling

**Figure 6:**
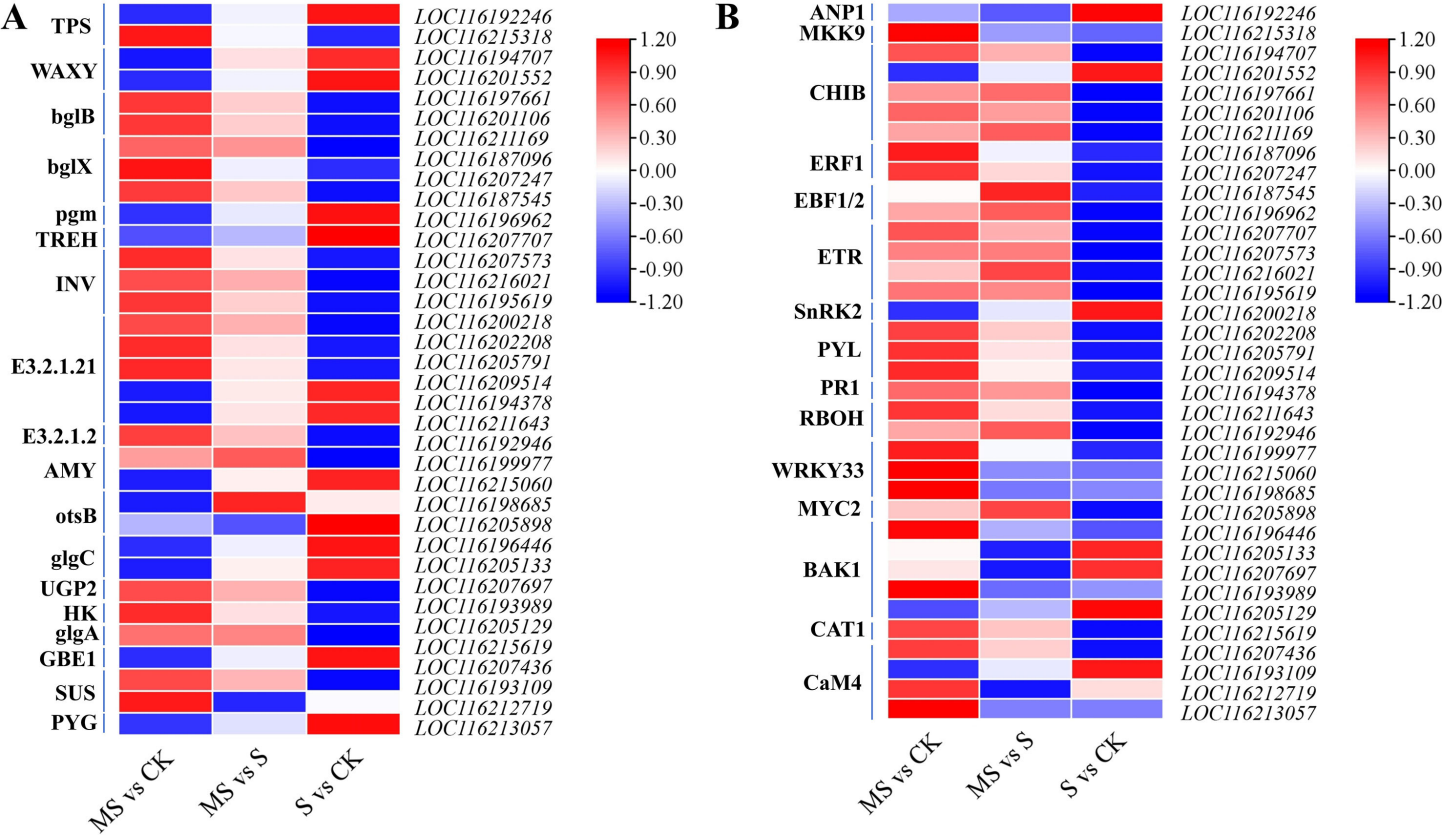
Effects of different treatments on starch and sucrose metabolic pathways and MAPK signaling. **(A)** Expression of DEGs in starch and sucrose metabolic signaling; **(B)** Expression of DEGs in MAPK signaling.

Salt stress significantly affected the expression of key genes in the starch and sucrose metabolic pathways in pomegranate seedlings (**Figure 6A**, **Supplementary Tables S6**, **S7**). Compared to the control, genes associated with starch degradation—including *ALPHA-AMYLASE* (*AMY*) and *STARCH PHOSPHORYLASE* (*PYG*)—are significantly up-regulated in the salt-treated group (S vs CK) (**Figure 6A**). Concurrently, the up-regulation of *GLYCOGEN BRANCHING ENZYME* (*GBE1*) is observed, collectively indicating a broad response of carbon metabolism pathways under salt stress. Melatonin application further modifies the expression patterns of genes related to starch and sucrose metabolism. In the melatonin-treated group (MS vs CK), *SUCROSE SYNTHASE* (*SUS*) and *URIDINE DIPHOSPHATE GLUCOSE PYROPHOSPHORYLASE* (*UGP2*) are up-regulated (**Figure 6A**). Moreover, the expression of *GLYCOGEN SYNTHASE* (*glgA*), a gene involved in starch synthesis that is suppressed under salt stress, is partially restored by melatonin treatment, suggesting a role for melatonin in regulating starch metabolic homeostasis.

With respect to the MAPK signaling pathway, salt stress markedly up-regulates the MAPKKK family gene *ARABIDOPSIS NUCLEUS- AND PHRAGMOPLAST-LOCALIZED PROTEIN KINASE 1* (*ANP1*) (**Figure 6B**), whereas its downstream MAPKK gene *MITOGEN-ACTIVATED PROTEIN KINASE KINASE 9* (*MKK9*) shows no significant change in expression in the S vs CK comparison. In the MS vs S comparison, the *PATHOGENESIS-RELATED PROTEIN 1* (*PR1*, *LOC116194378*) is strongly induced in the MS group, while the expression of the ethylene signaling hub gene *ETHYLENE RESPONSE FACTOR 1* (*ERF1*) is restored to near-baseline levels. Additionally, the *CALMODULIN 4* (*CaM4*) gene is specifically up-regulated under melatonin treatment (MS vs CK) (**Figure 6B**). These coordinated changes in gene expression suggest that melatonin may participate in the salt stress response in pomegranate seedlings by modulating the MAPK signaling pathway and associated defense-related genes.

### 3.8 DEGs associated with transcription factors

**Figure 7:**
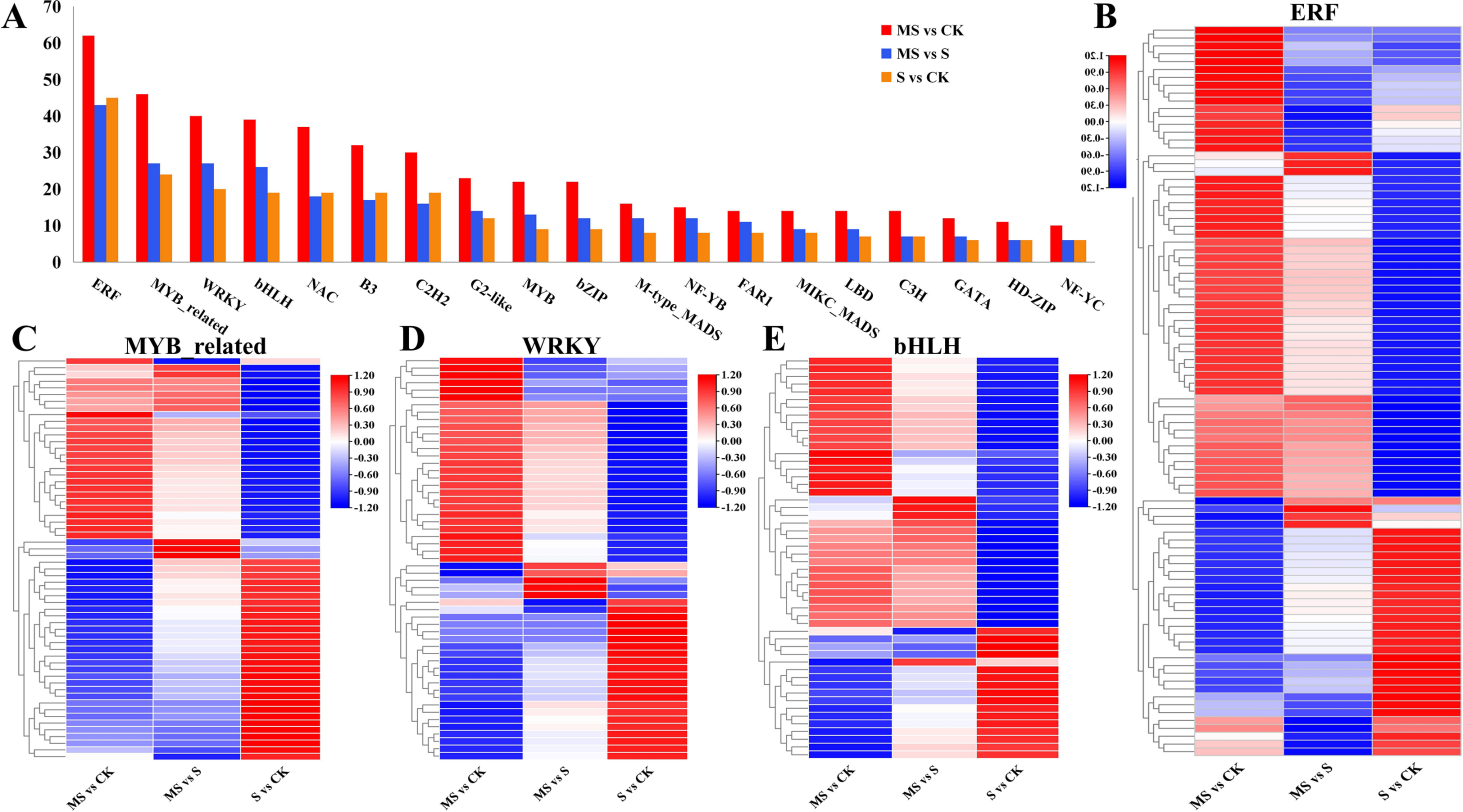
Effects of different treatments on pomegranate transcription factor (TF) families. **(A)** Number counts of key TF families under different treatments. **(B-E)** Heatmaps of differentially expressed genes (DEGs) in ERF, MYB_related, WRKY, and bHLH families.

Transcription factors usually regulate abiotic stresses in plants by mediating target genes. This study identified 1,276 TFs from 49 families that showed differential expression. The distributions of these TFs in various comparisons (MS vs CK, S vs CK, and MS vs S) were 599, 317, and 360, respectively (**Figure 7A**, **Supplementary Table S8**). Among the four comparative groups, ERF, MYB_related, WRKY, and bHLH families were the major enriched TFs, and their expression profiles are shown in **Figures 7B–E**. Further analysis by the investigators showed that three ERFs (two expressed down-regulation), four MYB_related (three expressed up-regulation and one expressed down-regulation), seven WRKYs (three expressed up-regulation and four expressed down-regulation) and six bHLH (three indicated up-regulation and three indicated down-regulation), whereas these genes only showed differential expression in MS vs S, suggesting that these genes are differentially expressed by the MT induction.

### 3.9 Joint physiological and transcriptomic analysis

**Figure 8:**
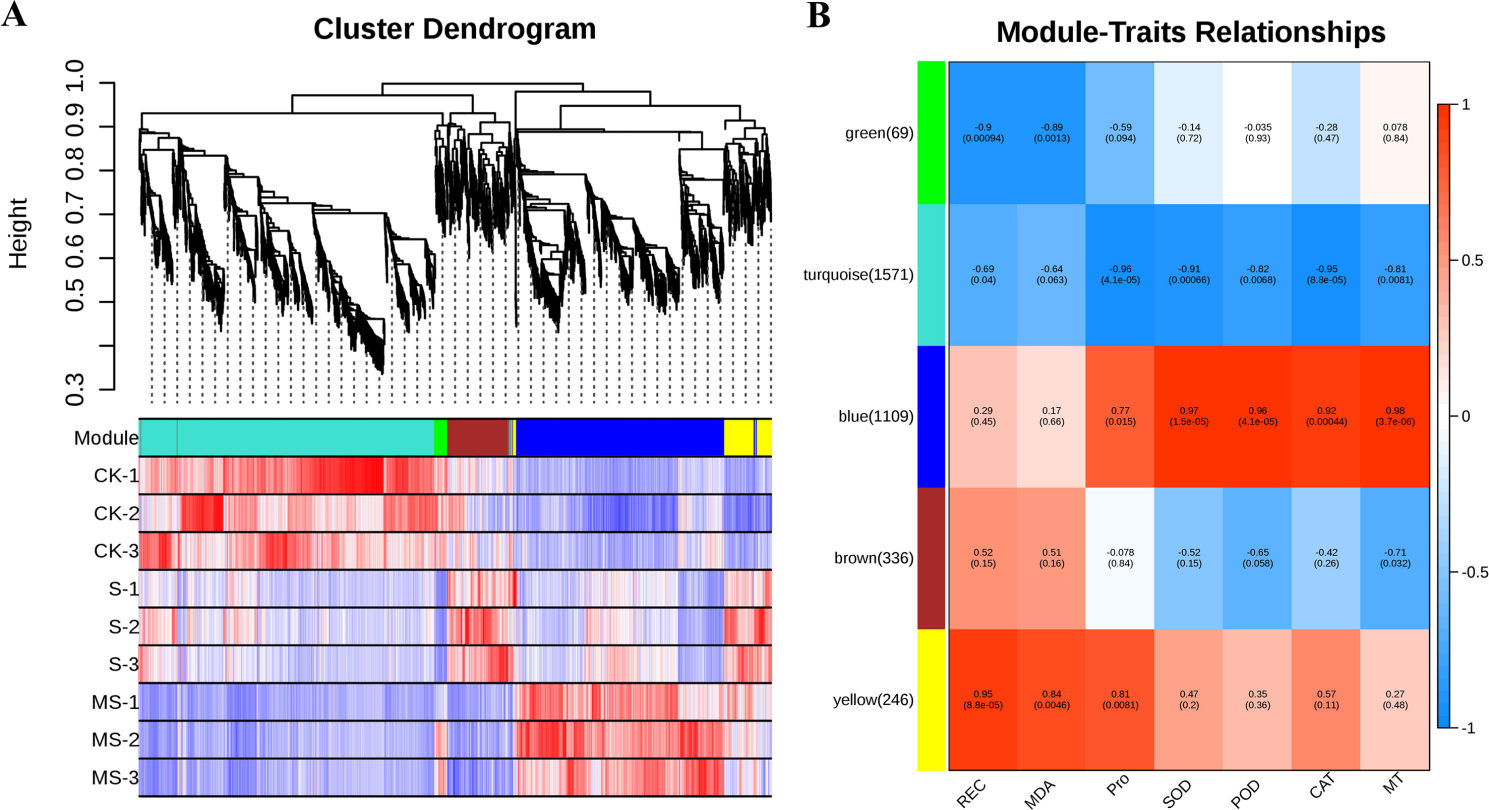
Co-expression module analysis. **(A)** Co-expression clusters identified by weighted gene co-expression network analysis (WGCNA). **(B)** Heatmap depicting correlations between modules and features. Each row represents a module, distinguished by a unique color (parentheses indicate the number of DEGs contained in the module). Each column is aligned to a specific feature. Red color indicates positive correlation and blue color indicates negative correlation.

Multiple genes control the response of pomegranate under different stresses. In this study, 3331 differential genes were identified by transcriptomic analysis of pomegranate seedlings under different treatments. The correlation between differential genes and physiological characteristics was further explored by WGCNA analysis. The analysis identified five gene co-expression modules (**Figures 8A, B**). The correlation analysis showed that the characteristics of SOD, POD, CAT, and MT content changes in the BLUE module were significantly and positively correlated with the gene expression levels, with correlation coefficients ranging from 0.92% to 0.98%. Meanwhile, SOD, POD, CAT, and MT content showed significant negative correlation with the turquoise module, with negative correlation coefficients ranging from 0.81%-0.96%. This finding suggests that genes in the blue module may play a role in pomegranate antioxidant capacity as well as MT-mediated salt tolerance. In addition, genes in the yellow module showed significant positive correlations with REC, MDA, and Pro content, suggesting that genes identified in this module may be involved in the regulation of scavenging of reactive oxygen species and maintenance of cellular membrane homeostasis. The blue module contained 1109 differential genes, and the yellow module contained 246 differential genes. Gene interactions within these two modules are shown in **Supplementary Table S9**, and three hub genes were identified based on kWithin values. Those in the yellow module include *LOC116203737*, *LOC116204633*, and *LOC116209845*. those in the blue module include *LOC116212144*, *LOC116200989*, and *LOC116213422*.

### 3.10 Real-time quantitative PCR analysis

**Figure 9:**
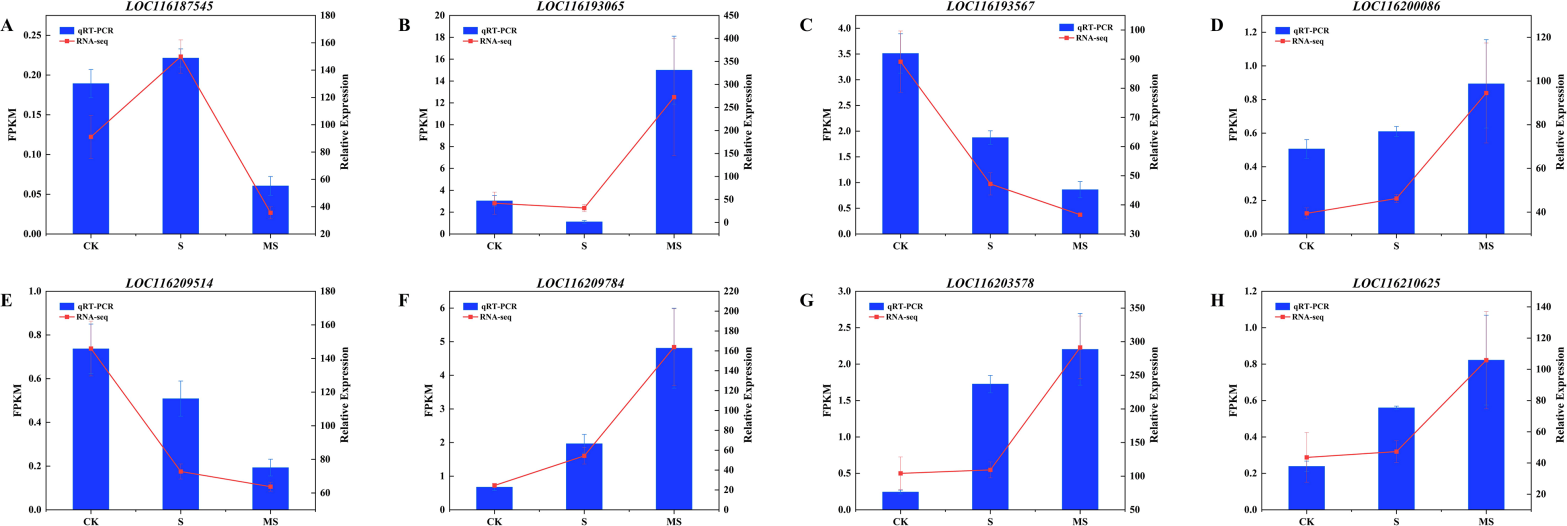
qRT-PCR results for 8 key genes. **(A-H)** (*LOC116187545*, *LOC116193065*, *LOC116193567*, *LOC116200086*, *LOC116209514*, *LOC116209784*, *LOC116203578* and *LOC116210625* genes). To ensure reproducibility and reliability, qRT-PCR analysis was performed on three independent biological replicates. Relative expression was calculated by the 2^-ΔΔCT^ method. Each experiment was repeated 3 times, and each sample in the experiments contained 3 biological replicates. The error bars in the graph represent standard deviation.

Eight differential genes were randomly selected to verify the accuracy of qRT-PCR RNA sequencing data. These DEGs included *LOC116187545*, *LOC116193065*, *LOC116193567*, *LOC116200086*, *LOC116203578*, *LOC116209514*, *LOC116209784*, and *LOC116210625*. qRT-PCR results showed that the candidate genes’ relative expression was consistent with the trend of transcript levels detected in the transcriptome data (**Figure 9**). This shows that the DEGs identified by RNA-seq are reliable and reproducible.

## 4 Discussion

### 4.1 Melatonin mitigates salt stress-induced photoinhibition by preserving photosynthetic apparatus integrity

Salt stress has been shown to have a significant impact on photosynthetic efficiency, primarily through the damage of the photosystem II (PSII) reaction center (Maxwell and Johnson, 2000). This reaction center is considered to be the component most vulnerable to environmental perturbation within the photosynthetic light reactions. In this study, treatment with 200 mM NaCl (S group) resulted in a significant reduction in the F_V_/F_M_ of pomegranate seedlings by 44.5% (**Figure 1A**), indicating severe impairment of primary photochemistry. Concurrently, the Y_II_ and qP decreased by 51% and 59%, respectively (**Figures 1B, C**), demonstrating substantial inhibition of photosynthetic electron transport and reduced energy allocation towards carbon assimilation. While NPQ increased by 134% (**Figure 1D**)—reflecting enhanced thermal dissipation to prevent excessive ROS accumulation—this represents significant energy wastage (Shi et al., 2021). These responses are consistent with chlorophyll fluorescence patterns observed in salt-stressed cucumber (*Cucumis sativus* L.) and willow (*Salix matsudana* Koidz) plants (Yuan et al., 2014; Ran et al., 2022), confirming the universal detrimental impact of salinity on the photosynthetic machinery.

The application of exogenous melatonin (400 μM, MS group) has been shown to effectively mitigate salt-induced damage to the photosynthetic apparatus through multiple mechanisms. Firstly, the structural integrity of the PSII reaction center is preserved: The F_V_/F_M_ ratio in the MS group was found to be 39.1% higher than in the S group (**Figure 1A**), indicating that primary photochemical efficiency has been maintained. This finding is consistent with reports of melatonin stabilizing PSII reaction center structure in tomato (Yang et al., 2023). Secondly, the allocation of light energy allocation is optimized, thereby reducing inefficient thermal dissipation. Y_II_ and qP increased by 32.2% and 41.8%, respectively, in the MS group compared to the S group (**Figures 1B, C**), demonstrating improved energy utilization for photochemistry and carbon assimilation. Concurrently, NPQ is reduced by 45% in the MS group relative to the S group (**Figure 1D**), indicating a rebalancing of non-photochemical quenching processes and prevention of excessive energy dissipation (Yang et al., 2023). While NPQ induction under salt stress protects against ROS accumulation, its overactivation leads to wasteful energy loss (Shi et al., 2021); melatonin, by moderating NPQ, facilitates greater energy flux towards CO_2_ fixation, thereby enhancing photosynthetic efficiency (Yang et al., 2023). Furthermore, thylakoid membrane stability is maintained, preventing lipid peroxidation. As the functional matrix for the PSII reaction center, thylakoid membrane integrity is essential for photosynthesis (Ran et al., 2022). The significantly lower MDA content in the MS group compared to the S group (**Figure 1D**) confirms that melatonin suppresses membrane lipid peroxidation, likely via ROS scavenging (Gill and Tuteja, 2010), thereby providing a stable microenvironment for PSII function.

### 4.2 Salt tolerance enhancement by melatonin through coordinated regulation of the antioxidant system, osmotic homeostasis, and carbon metabolism reprogramming

Under salt stress, plants are challenged by oxidative stress (ROS overaccumulation) and osmotic imbalance (cellular dehydration induced by ion toxicity). The combined effect of these stressors is to synergistically exacerbate membrane lipid peroxidation (elevated MDA) and increase membrane permeability (elevated REC), ultimately resulting in growth suppression (Hasanuzzaman et al., 2020; Zulfiqar et al., 2024). The findings of this study demonstrate that exogenous melatonin application is associated with a salt stress mitigation phenotype, involving the activation of the antioxidant system, accumulation of osmoprotectants, and adjustment of carbon metabolism pathways. The integration of physiological indicators with transcriptomic data provides valuable insights into the potential molecular mechanisms through which melatonin enhances salt tolerance.

Salt stress significantly induced ROS accumulation in pomegranate seedlings (Waheed et al., 2024), resulting in a 0.43-fold increase in MDA content and a 0.46-fold increase in REC compared to the CK group (**Figures 2D, E**). This response is consistent with reports in salt-stressed *Cornus hongkongensis* subsp. (Yuan et al., 2022) and *Gossypium hirsutum* L (Li et al., 2022). However, MT treatment has been shown to significantly reduce ROS levels (**Figures 2A–C**) and stabilize membrane structure, as indicated by 16.50% and 18.1% reductions in MDA and REC, respectively, in comparison to the S group. This protection is achieved through the synergistic activation of antioxidant enzyme activities: SOD, POD, and CAT are elevated by 14.3%, 21.7%, and 11.7%, respectively, relative to the S group. At the transcriptional level, antioxidant-related genes such as *CATALASE 1* (*CAT1*, *LOC116215619*) and *RESPIRATORY BURST OXIDASE HOMOLOG* (*RBOH*, *LOC116211643*, *LOC116192946*) are significantly up-regulated in the MS group (**Figure 6B**), suggesting that gene expression regulation may underpin the enhancement of the antioxidant system (Arnao and Hernández-Ruiz, 2019). Furthermore, endogenous melatonin levels in the MS group are 63.1% higher than those in the S group (**Figure 3A**). Exogenous MT likely acts in concert with the endogenous system to reinforce antioxidant defense, possibly via the up-regulation of key biosynthesis genes such as *SEROTONIN N-ACETYLTRANSFERASE* (*SNAT*) and *ACETYLSEROTONIN O-METHYLTRANSFERASE* (*ASMT*) (Byeon et al., 2014).

Carbon metabolism plays an essential role in maintaining osmotic homeostasis. Under salt stress, the accumulation of Pro (**Figure 2F**) and the up-regulation of starch degradation genes such as *STARCH PHOSPHORYLASE* (*PYG*, **Figure 5A**) indicate that plants may mobilize stored carbon sources and accumulate soluble compounds to cope with osmotic stress. This strategy bears a resemblance to the MT-mediated mechanism in alfalfa (*Medicago sativa*), in which α-amylase activation promotes carbon release (Guo et al., 2025). Notably, melatonin treatment further enhances the expression of sucrose synthesis genes *SUS* and *UGP2* (**Figure 6A**), while the starch synthase gene, which is down-regulated in the S group, is partially restored in the MS group (**Figure 6A**). Collectively, these expression patterns suggest that melatonin may not only promote sucrose synthesis to improve osmotic adjustment but also help maintain starch metabolic homeostasis, thereby preventing excessive carbon consumption (Jannatizadeh, 2019). While similar metabolic regulation has been reported in rice (Chen et al., 2021), the distinct response of *glgA* in pomegranate may reflect species-specific metabolic adaptation strategies.

In summary, during the melatonin-mediated enhancement of salt tolerance, the reinforcement of the antioxidant system helps maintain membrane integrity, thereby providing a foundation for cellular osmotic adjustment. Meanwhile, the regulation of carbon metabolism-related gene expression likely supplies carbon skeletons and energy support for the synthesis of compatible solutes such as proline. The coordinated operation of these physiological processes may constitute a key basis for the improved salt adaptation capacity observed in pomegranate seedlings.

### 4.3 Multi-pathway salt tolerance regulation by melatonin through hormone-MAPK synergistic network

The MAPK (Mitogen Activated Protein Kinase) signaling pathway is a key signaling network in plant response to salt stress, which converts external stress signals into intracellular physiological responses through a three-stage kinase cascade (MAPKKK-MAPKK-MAPK) (Guo et al., 2025). In pomegranate seedlings, salt stress significantly up-regulates the MAPKKK family gene *ANP1* (*LOC116192246*) (**Figure 6B**). However, its downstream MAPKK gene *MKK9* (*LOC116215318*) shows no significant change in expression, suggesting that salt stress may regulate this signaling cascade at the post-transcriptional level. This phenomenon is similar to the mechanism by which salt stress inhibits *MPK6* activity through ubiquitination modifications in Arabidopsis (*Arabidopsis thaliana*) (Yu et al., 2010). In the melatonin-treated group, up-regulation of genes such as *WRKY DNA-BINDING PROTEIN 33* (*WRKY33*) and *PATHOGENESIS-RELATED PROTEIN 1* (*PR1*) is observed, indicating a potential functional linkage between MAPK signaling and the antioxidant defense system (Mittler et al., 2011). The regulatory scope of the MAPK pathway is further expanded through its interplay with calcium signaling. In this study, the calmodulin gene *CALMODULIN 4* (*CaM4*, *LOC116213057*) is significantly up-regulated in the MS group (**Figure 6B**), suggesting that calcium ions may participate in modulating the activity of the ANP1–MKK9–MAPK module, thereby collectively influencing salt tolerance. A comparable mechanism has been documented in rice (*Oryza sativa*), where *CALCIUM-DEPENDENT PROTEIN KINASE* (*CDPK*) modulates salt tolerance by phosphorylating the MAPKKK protein *OsMKKK70* and activating the OsMPK3/6 cascade signaling pathway (Su et al., 2024).

Plant hormone networks have been demonstrated to exhibit highly dynamic regulatory characteristics in response to salt stress (Khan et al., 2022), while melatonin has been shown to markedly enhance salt tolerance by modulating hormone homeostasis (Zhu et al., 2024b). In the MS group, the core salt-stress signaling molecule ABA and its receptors *PYRABACTIN RESISTANCE 1-LIKE PROTEIN* (*PYL*) are significantly up-regulated (**Figure 5**), suggesting that melatonin may contribute to the establishment of osmotic balance by regulating ABA signaling. The activation of SA signaling further underscores the synergistic effects of melatonin across various pathways. The marked up-regulation of *NPR1* and *PR1* in the MS group (**Figure 5**) is closely linked to the activation of the *MAPK-WRKY33* module. In tomato plants, the *MPK3/6-WRKY33* axis facilitates the expression of the *PR1* promoter by directly binding to it, thereby enhancing disease resistance (Meng and Zhang, 2013). In the present study, *WRKY33* was found to be up-regulated in the MS group (**Figure 6B**), suggesting that it may synergistically modulate SA signaling and reactive oxygen species (ROS) metabolism through a similar mechanism.

In summary, melatonin is likely involved in the salt stress adaptation of pomegranate seedlings by regulating key components of the MAPK cascade and engaging in a complex interaction network with calcium signaling and multiple phytohormone pathways.

### 4.4 Melatonin-regulated co-expression network reveals antioxidant-membrane stability association

By weighted gene co-expression network analysis (WGCNA), this study identified that the blue module was significantly positively correlated with antioxidant capacity (SOD, CAT) and MT content (**Figure 8B**), and its core genes, such as *LOC116212144*, may be directly involved in salt tolerance through the regulation of ROS metabolism. In addition, the yellow module was found to be positively correlated with the indicators of membrane damage indicators (MDA, REC) were positively correlated, and its pivotal gene, *LOC116203737*, is speculated to play a role in membrane repair or lipid peroxidation inhibition (Porta and Rocha-Sosa, 2002). These results not only validate the reliability of the transcriptomic data, but also provide new perspectives for resolving the genetic basis of complex traits. It should be noted that this study is primarily based on physiological and transcriptomic analyses. While our data provide strong correlative evidence for MT’s role in enhancing salt tolerance and reveal multiple key genes and pathways, the precise molecular mechanisms and causal relationships require further functional validation.

## 5 Conclusion

Through the integration of physiological phenotyping and transcriptomic analysis, this study reveals the potential role of exogenous MT in mitigating salt stress damage in pomegranate seedlings. The results demonstrate that MT treatment is closely associated with improved photosynthetic performance under salt stress, as indicated by increased F_V_/F_M_ and Y_II_ of PSII, elevated qP, and reduced NPQ. At the physiological level, MT application correlates with enhanced activities of antioxidant enzymes (SOD, POD, CAT), decreased content of the membrane lipid peroxidation product MDA, and improved membrane stability (reduced REC). Transcriptome data further reveal that MT treatment is linked to extensive expression changes in genes involved in carbon metabolism (e.g., *SUS*, *UGP2*, *glgA*), the MAPK signaling pathway (e.g., *ANP1*, *MKK9*), and plant hormone signal transduction. WGCNA identifies key modules and hub genes (e.g., *LOC116212144* and *LOC116203737*) significantly associated with antioxidant traits and membrane stability. In summary, exogenous melatonin likely enhances salt tolerance in pomegranate seedlings by coordinating antioxidant defense, maintaining photosynthetic function, modulating carbon metabolism, and influencing multiple stress-related signaling pathways. This study provides important data and theoretical insights for elucidating the mechanisms of melatonin in the salt stress response of woody plants.

## Data Availability

The dataset supporting the findings of this study is available in the NCBI BioProject database under accession number PRJNA1285076 (https://www.ncbi.nlm.nih.gov/bioproject/PRJNA1285076).

## References

[B1] Arnao M. B. Hernández-Ruiz J. (2019). Melatonin: A new plant hormone and/or a plant master regulator? Trends Plant Sci. 24, 38–48. doi: 10.1016/j.tplants.2018.10.010, PMID: 30446305

[B2] Byeon Y. Lee H. Y. Lee K. Back K. (2014). Caffeic acid O-methyltransferase is involved in the synthesis of melatonin by methylating N-acetylserotonin in Arabidopsis. J. Pineal Res. 57, 219–227. doi: 10.1111/jpi.12160, PMID: 25039887

[B3] Cai H. Li J. Li J. Teng H. (2025). Melatonin—Angel of plant growth regulation and protection. Advanced Agrochem. 4, 114-122. doi: 10.1016/j.aac.2025.01.001

[B4] Chen Y. Li R. Ge J. Liu J. Wang W. Xu M.. (2021). Exogenous melatonin confers enhanced salinity tolerance in rice by blocking the ROS burst and improving Na+/K+ homeostasis. Environ. Exp. Bot. 189, 104530. doi: 10.1016/j.envexpbot.2021.104530

[B5] Gill S. S. Tuteja N. (2010). Reactive oxygen species and antioxidant machinery in abiotic stress tolerance in crop plants. Plant Physiol. Biochem. 48, 909–930. doi: 10.1016/j.plaphy.2010.08.016, PMID: 20870416

[B6] Guo S. Wang X. Li X. Ma Y. Yang J. Fu B.. (2025). Melatonin and calcium synergistically improve salt tolerance in alfalfa (*Medicago sativa.* L). Ind. Crops Products 224, 120322. doi: 10.1016/j.indcrop.2024.120322

[B7] Hao X. Sun B. Song Y. Zhang J. Wu J. Zhang N.. (2024). Melatonin-mediated physiological and molecular responses to abiotic stress in horticultural crops. Hortic. Plant J. 11, 1381-1396. doi: 10.1016/j.hpj.2024.08.006

[B8] Hasanuzzaman M. Bhuyan M. H. M. B. Zulfiqar F. Raza A. Mohsin S. M. Mahmud J. A.. (2020). Reactive oxygen species and antioxidant defense in plants under abiotic stress: revisiting the crucial role of a universal defense regulator. Antioxidants (Basel) 9, 681. doi: 10.3390/antiox9080681, PMID: 32751256 PMC7465626

[B9] Himanshu Sharma S. Rana V. S. Ankit Thakur V. Kumar A.. (2024). Unlocking the sustainable role of melatonin in fruit production and stress tolerance: a review. CABI Agric. Bioscience 5, 103. doi: 10.1186/s43170-024-00309-z

[B10] Jannatizadeh A. (2019). Exogenous melatonin applying confers chilling tolerance in pomegranate fruit during cold storage. Scientia Hortic. 246, 544–549. doi: 10.1016/j.scienta.2018.11.027

[B11] Khan Z. Jan R. Asif S. Farooq M. Jang Y.-H. Kim E.-G.. (2024). Exogenous melatonin induces salt and drought stress tolerance in rice by promoting plant growth and defense system. Sci. Rep. 14, 1214. doi: 10.1038/s41598-024-51369-0, PMID: 38216610 PMC10786868

[B12] Khan T. A. Saleem M. Fariduddin Q. (2022). Recent advances and mechanistic insights on Melatonin-mediated salt stress signaling in plants. Plant Physiol. Biochem. 188, 97–107. doi: 10.1016/j.plaphy.2022.08.007, PMID: 35995025

[B13] Li W.-Q. Li J.-Y. Bi S.-J. Jin J.-Y. Fan Z.-L. Shang Z.-L.. (2025). Melatonin enhances maize germination, growth, and salt tolerance by regulating reactive oxygen species accumulation and antioxidant systems. Plants 14, 296. doi: 10.3390/plants14020296, PMID: 39861647 PMC11768311

[B14] Li L. Qi Q. Zhang H. Dong Q. Iqbal A. Gui H.. (2022). Ameliorative Effects of Silicon against Salt Stress in Gossypium hirsutum L. Antioxidants 11, 1520. doi: 10.3390/antiox11081520, PMID: 36009240 PMC9404900

[B15] Liu X. Du L. Yang X. Yin B. Wang L. Wang Y. (2023). Physicochemical properties of Tunisian pomegranate fruits Punica granatum L. grown at different climatic zones of Yunnan, China. Heliyon 9, e14791. doi: 10.1016/j.heliyon.2023.e14791, PMID: 37035371 PMC10073889

[B16] Liu C. Zhao X. Yan J. Yuan Z. Gu M. (2019). Effects of salt stress on growth, photosynthesis, and mineral nutrients of 18 pomegranate (Punica granatum) cultivars. Agronomy 10, 27. doi: 10.3390/agronomy10010027

[B17] Livak K. J. Schmittgen T. D. (2001). Analysis of relative gene expression data using real-time quantitative PCR and the 2–ΔΔCT method. Methods 25, 402–408. doi: 10.1006/meth.2001.1262, PMID: 11846609

[B18] Maxwell K. Johnson G. N. (2000). Chlorophyll fluorescence—a practical guide. J. Exp. Bot. 51, 659–668. doi: 10.1093/jexbot/51.345.659 10938857

[B19] Meng X. Zhang S. (2013). MAPK cascades in plant disease resistance signaling. Annu. Rev. Phytopathol. 51, 245–266. doi: 10.1146/annurev-phyto-082712-102314, PMID: 23663002

[B20] Mittler R. Vanderauwera S. Suzuki N. Miller G. Tognetti V. B. Vandepoele K.. (2011). ROS signaling: the new wave? Trends Plant Sci. 16, 300–309. doi: 10.1016/j.tplants.2011.03.007, PMID: 21482172

[B21] Nawaz M. Sun J. Shabbir S. Khattak W. A. Ren G. Nie X.. (2023). A review of plants strategies to resist biotic and abiotic environmental stressors. Sci. Total Environ. 900, 165832. doi: 10.1016/j.scitotenv.2023.165832, PMID: 37524179

[B22] Porta H. Rocha-Sosa M. (2002). Plant lipoxygenases. Physiological and molecular features. Plant Physiol. 130, 15–21. doi: 10.1104/pp.010787, PMID: 12226483 PMC1540254

[B23] Ran X. Wang X. Huang X. Ma C. Liang H. Liu B. (2022). Study on the relationship of ions (Na, K, ca) absorption and distribution to photosynthetic response of Salix matSudana Koidz under salt stress. Front. Plant Sci. 13. doi: 10.3389/fpls.2022.860111, PMID: 35592567 PMC9111522

[B24] Samanta S. Roychoudhury A. (2023). Crosstalk of melatonin with major phytohormones and growth regulators in mediating abiotic stress tolerance in plants. South Afr. J. Bot. 163, 201–216. doi: 10.1016/j.sajb.2023.10.040

[B25] Shi C. Yang F. Liu Z. Li Y. Di X. Wang J.. (2021). Uniform water potential induced by salt, alkali, and drought stresses has different impacts on the seedling of Hordeum jubatum: from growth, photosynthesis, and chlorophyll fluorescence. Front. Plant Sci. 12. doi: 10.3389/fpls.2021.733236, PMID: 34659299 PMC8514703

[B26] Singh A. Mann A. Kumar R. Yadav R. K. (2023). Delineating eco-physiological traits linked to salt tolerance and fruit yield in pomegranate. Scientia Hortic. 322, 112422. doi: 10.1016/j.scienta.2023.112422

[B27] Sinha N. Chaudhary S. Rani R. Karuna K. Ahmad M. (2022). Impact of salt stress on fruits and its mitigation strategies; A review. Ann. Plant Sci. 11, 4692–4704. doi: 10.21746/aps.2022.11.01.17

[B28] Su S. Jiang Y. Zhu X. Yu S. Wang F. Xue L.. (2024). Calcium-dependent protein kinases 5 and 13 enhance salt tolerance in rice by directly activating OsMPK3/6 kinases. Plant Physiol. 196, 3033–3047. doi: 10.1093/plphys/kiae520, PMID: 39361658 PMC11638333

[B29] Waheed A. Zhuo L. Wang M. Hailiang X. Tong Z. Wang C.. (2024). Integrative mechanisms of plant salt tolerance: Biological pathways, phytohormonal regulation, and technological innovations. Plant Stress 14, 100652. doi: 10.1016/j.stress.2024.100652

[B30] Wang J. Ao H. Zhang J. (2003). Experimental techniques and principles of plant physiology and biochemistry (Harbin: Northeast Forestry University Press).

[B31] Xiao W. (2020). Experimental supervision of plant physiology (Guangzhou: Sun Yat-sen University Press).

[B32] Xie X. Ding D. Bai D. Zhu Y. Sun W. Sun Y.. (2022). Melatonin biosynthesis pathways in nature and its production in engineered microorganisms. Synthetic Syst. Biotechnol. 7, 544–553. doi: 10.1016/j.synbio.2021.12.011, PMID: 35087957 PMC8761603

[B33] Yan F. Wei H. Ding Y. Li W. Chen L. Ding C.. (2021). Melatonin enhances Na+/K+ homeostasis in rice seedlings under salt stress through increasing the root H+-pump activity and Na+/K+ transporters sensitivity to ROS/RNS. Environ. Exp. Bot. 182, 104328. doi: 10.1016/j.envexpbot.2020.104328

[B34] Yang Y. Guan S. Jiang X. Li M. Wei S. Diao M. (2023). Exogenous melatonin alleviates the inhibitory effect of naHCO3 on tomato growth by regulating the root pH value and promoting plant photosynthesis. Agronomy 13, 2777. doi: 10.3390/agronomy13112777

[B35] Yu L. Nie J. Cao C. Jin Y. Yan M. Wang F.. (2010). Phosphatidic acid mediates salt stress response by regulation of MPK6 in Arabidopsis thaliana. New Phytol. 188, 762–773. doi: 10.1111/j.1469-8137.2010.03422.x, PMID: 20796215

[B36] Yuan Y. Shu S. Li S. He L. Li H. Du N.. (2014). Effects of exogenous putrescine on chlorophyll fluorescence imaging and heat dissipation capacity in cucumber (Cucumis sativus L.) under salt stress. J. Plant Growth Regul. 33, 798–808. doi: 10.1007/s00344-014-9427-z

[B37] Yuan J.-Q. Sun D.-W. Lu Q. Yang L. Wang H.-W. Fu X.-X. (2022). Responses of Physiology, Photosynthesis, and Related Genes to Saline Stress in Cornus hongkongensis subsp. tonkinensis (W. P. Fang) Q. Y. Xiang. Plants (Basel) 11, 940. doi: 10.3390/plants11070940, PMID: 35406920 PMC9002922

[B38] Zhang N. Sun Q. Zhang H. Cao Y. Weeda S. Ren S.. (2015). Roles of melatonin in abiotic stress resistance in plants. J. Exp. Bot. 66, 647–656. doi: 10.1093/jxb/eru336, PMID: 25124318

[B39] Zhou H. Shi H. Yang Y. Feng X. Chen X. Xiao F.. (2024). Insights into plant salt stress signaling and tolerance. J. Genet. Genomics 51, 16–34. doi: 10.1016/j.jgg.2023.08.007, PMID: 37647984

[B40] Zhu M. Guo T. Liu Y. B. Xiao R. Yu T. Huang J. X.. (2024a). Ascorbic acid is involved in melatonin-induced salinity tolerance of maize (Zea mays L.) by regulating antioxidant and photosynthetic capacities. Photosynthetica 62, 361–371. doi: 10.32615/ps.2024.039, PMID: 39811709 PMC11726288

[B41] Zhu Y. Pal S. Xia X. (2024b). The role of hormones in plant stress: The old and new players. Plant Stress 13, 100552. doi: 10.1016/j.stress.2024.100552

[B42] Zhuang Q. Shao Z. Huang X. Zhang Y. Wu W. Feng X.. (2021). Evolution of soil salinization under the background of landscape patterns in the irrigated northern slopes of Tianshan Mountains, Xinjiang, China. CATENA 206, 105561. doi: 10.1016/j.catena.2021.105561

[B43] Zulfiqar F. Nafees M. Moosa A. Ferrante A. Darras A. (2024). ). Melatonin induces proline, secondary metabolites, sugars and antioxidants activity to regulate oxidative stress and ROS scavenging in salt stressed sword lily. Heliyon 10, e32569. doi: 10.1016/j.heliyon.2024.e32569, PMID: 38961974 PMC11219490

